# Therapeutic effects of axitinib, an anti-angiogenic tyrosine kinase inhibitor, on interstitial cystitis

**DOI:** 10.1038/s41598-023-35178-5

**Published:** 2023-05-23

**Authors:** Jung Hyun Shin, Chae-Min Ryu, Hwan Yeul Yu, Yang Soon Park, Dong-Myung Shin, Myung-Soo Choo

**Affiliations:** 1 Department of Urology, Ewha Womans University Mokdong Hospital, Seoul, Korea; 2 Department of Urology, Asan Medical Center, Ulsan University College of Medicine, Seoul, Korea; 3 Center for Cell Therapy, Asan Medical Center, Ulsan University College of Medicine, Seoul, Korea; 4 Department of Pathology, Asan Medical Center, Ulsan University College of Medicine, Seoul, Korea; 5 Department of Cell and Genetic Engineering, Asan Medical Center, Ulsan University College of Medicine, Seoul, Korea

**Keywords:** Medical research, Urology

## Abstract

To investigate the therapeutic effects of axitinib, a tyrosine kinase inhibitor, in an interstitial cystitis (IC) rat model. IC patients with or without Hunner lesion and non-IC controls were enrolled (n = 5/group). Bladder tissues were stained with vascular endothelial growth factor (VEGF), VEGF receptor 2 (VEGFR-2), platelet-derived growth factor (PDGF), and PDGF receptor B (PDGFR-B). The IC group showed extensive VEGFR-2 and PDGFR-B staining compared with controls. Next, ten-week-old female Sprague Dawley rats were divided into three groups (n = 10/group): sham, hydrochloride (HCl), and axitinib groups. One week after HCl instillation (day 0), the axitinib group received oral axitinib (1 mg/kg) for five consecutive days and pain was evaluated daily. Bladder function, histology and genetics were evaluated on day 7. The pain threshold significantly improved 3 days after axitinib administration. Axitinib decreased non-voiding contraction and increased the micturition interval and micturition volume and alleviated urothelial denudation, angiogenesis, mast cell infiltration, and fibrosis. HCl instillation increased the expression of tyrosine kinase receptors, including VEGFR-2 and PDGFR-B; axitinib administration inhibited their expression. Oral administration of axitinib improved pain, voiding profiles, and urothelial integrity by inhibiting angiogenesis in IC rat model. Axitinib may have potential therapeutic efficacy in IC patients.

## Introduction

Interstitial cystitis/bladder pain syndrome (IC/BPS) is characterized by chronic pelvic pain provoked by bladder filling and usually accompanies lower urinary tract symptoms such as frequency, urgency, and nocturia^1,2^. Various mechanisms have been suggested to explain the pathogenesis of IC, which include mast cell activation, gag layer damage, potassium hypersensitivity, and autoimmunity; however, pathophysiology of this disease is yet to be determined^3^. IC/BPS is a chronic inflammatory disease that is considered to be loco-extensive and affects the whole bladder^4^. The presence of Hunner lesions on cystoscopy can classify IC/BPS into Hunner type (IC) and non-Hunner type (BPS); current guidelines suggest that fulguration of the Hunner lesion is recommended for IC patients^2,5^.

Several studies have reported that the bladder tissue of IC/BPS patients presents with significantly higher expression of VEGF (vascular endothelial growth factor) than healthy control tissue, resulting in increased immature angiogenesis^6,7^. VEGF is important in maintaining tight cell junctions and bladder permeability; it is not only expressed in the bladder blood vessels, but also in apical cells and intramural ganglia^8^. Axitinib is a tyrosine kinase inhibitor assumed to primarily act on VEGF receptor (VEGFR) 1–3 and platelet-derived growth factor receptor (PDGFR) to inhibit angiogenesis and cell proliferation. Axitinib is currently indicated for the treatment of metastatic renal cell carcinoma^9^.

Our study hypothesis is as follows: if IC/BPS bladders present with increased expression of VEGF, agents that affect VEGF or VEGFR expression/activation would have a therapeutic effect in such patients. In this study, we initially examined the bladder tissues of IC/BPS patients to assess changes in the expression of angiogenic growth factors (VEGF and PDGF) and their tyrosine receptor kinases (VEGFR and PDGFR). Next, we investigated the therapeutic effects of axitinib in a hydrochloride (HCl)-induced IC rat model.

## Materials and methods

### Ethics statement

The Institutional Review Board of the Asan Medical Center approved the use of human bladder tissue (IRB No. 2021-1154). Informed consent was acquired from all participants prior to the tissue analysis and the whole process for human tissue analysis was performed in accordance with the relevant guidelines and regulations. The animal experiment was approved and performed in accordance with the guidelines and regulations of the Institutional Animal Care and Use Committee of University of Ulsan, College of Medicine (IACUC No. 2019-12-309). This study was performed in accordance with ARRIVE guidelines.

### Human bladder tissue analysis

Patients who underwent transurethral resection and cauterization of Hunner lesion (n = 5) for IC and hydrodistension (n = 5) for BPS were enrolled, with an additional five non-IC/BPS patients as the control group. Bladder tissue was obtained by either using an electrical loop or cold cup biopsy. The diagnosis of IC/BPS was based on the American Urological Association criteria. The bladder tissue of included patients was stained for VEGF (1:200, mouse monoclonal, clone G153-694, catalogue no.555036, Parmingen, New Jersey, US), VEGFR-2 (1:400, rabbit monoclonal, clone 55B11, catalogue no.2479, CST, Massachusetts, USA), PDGF (1:200, rabbit monoclonal, clone L48, Catalogue No. BS1290, Bioworld, MN, USA), and PDGFR-B (1:200, rabbit monoclonal, clone Y92, Catalogue No. 1469-1, Epitomics, CA, USA). The histological examination was performed by a single urological pathologist who was blinded to the clinical information. The positive staining area was analyzed with the HALO® IMAGE ANALYSIS PLATFORM (Version 3.2, Indica Labs).

### Animal modelling

To induce acute urothelial injury, 10-week-old female Sprague Dawley rats (OrientBio, Seongnam, Gyeonggi-do, Korea) received intravesical instillation of 0.2 M HCl via transurethral insertion of a 26-gauge angiocatheter. After 10 min, the bladder was emptied and washed with normal saline. Phosphate-buffered saline (PBS) solution was used instead of HCl in the sham (control) group.

### Administration of axitinib and pain evaluation

Ten-week-old female Sprague Dawley rats were divided into three groups: sham (n = 5), HCl group (n = 5), and axitinib group (n = 5). One week after instillation (day 0), the axitinib group received oral administration of axitinib (Pfizer, 1 mg/kg) for 5 days (day 1–day 5), with a 2-day rest period. During axitinib administration, the manual von Frey test (Touch Test™ Sensory Evaluators; 58,011, Stoelting, IL, USA) was performed daily for pain evaluation. The initial stimulus was a 10 g (diameter: 5.07) filament. If there was no response, the next higher force filament was tested; if there was a response, the next filament with a lower force was tested. The test was continued until at least four records were obtained after the first change of response direction (positive to negative response or negative to positive response).

### Bladder function evaluation

Two weeks after instillation (day 7), awake cystometry was performed to evaluate bladder function. The definitions of the parameters used for analysis are as follows: non-voiding contraction was an increase in intravesical pressure above 15 cmH_2_O from baseline without a recorded voiding volume; the micturition interval was the interval between each voiding; the micturition volume was the amount of voided urine recorded by a fluid collector; bladder capacity was the total amount of infused saline; and micturition pressure was the maximum detrusor pressure during the voiding phase. The mean values from three reproducible voiding cycles in individual animals were used for analysis.

### Histological and genetic analysis

After awake cystometry, the bladder was harvested for histological and genetic expression analysis. Histological analysis was performed to evaluate epithelial denudation, vessels, mast cell infiltration, tissue fibrosis, and apoptosis with cytokeratin immunostaining (Keratin, Pan Ab-1; Thermo Scientific, MA, USA), CD31 staining (sc-376764; Santa Cruz Biotechnology, TX, USA), toluidine blue staining (Toluidine blue-O; Daejung Chemicals & Metals, Seoul, Korea), Masson’s trichrome staining (Junsei Chemical, Tokyo, Japan), VEGFR2 (#2472, Cell Signaling Technology, Danvers, MA, USA) and PDGFR-α (sc-398246; Santa Cruz Biotechnology, Dallas, TX, USA) respectively. Quantitative digital image analysis was performed in two randomly selected representative areas of each slide from five independent animals with Image-Pro 5.0 software (Media Cybernetics, Rockville, MD, USA). The PDGF and VEGF associated genes were quantified by real-time quantitative polymerase chain reaction (RQ-PCR) analysis^10^. The overall shematic descripton of the experiment is graphically summarized in Supplementary Fig. 1.

### Statistical analysis

The clinical parameters were analyzed with the chi-square test for categorical variables and the Mann–Whitney test for non-parametric variables using SPSS version 21.0. Other data were reported as mean ± standard error of the mean (SEM) and were analyzed with GraphPad Prism 7.0 (GraphPad Software, La Jolla, CA, USA). Differences and significance were verified using one-way or two-way ANOVA followed by Bonferroni post-hoc tests. *P* values of < 0.05 were considered statistically significant.

## Results

### Expression of VEGF and PDGF in human bladder tissues

Bladder tissues from a total of fifteen patients with a mean age of 64.5 ± 10.0 years were included in the analysis (IC; n = 5, BPS; n = 5, control; n = 5). There were no significant differences in baseline symptom scores based on validated questionnaires between the IC and BPS groups (Table 1).

**Table 1:** Baseline demographics of patients.

	IC (n = 5)	BPS (n = 5)	Control (n = 5)	*P* value
Age (years)	68.4 ± 13.4	58.8 ± 8.1	66.4 ± 6.3	0.222
Sex (M:F)	1:4	2:3	2:3	0.741
Symptom duration (months)	41.7 ± 61.6	33.3 ± 27.7	N/A	1.000†
Baseline symptom				
VAS	5.4 ± 2.6	7.4 ± 1.3	N/A	0.151†
ICIS	13.8 ± 4.0	14.6 ± 2.9	N/A	0.548†
ICIP	10.0 ± 3.7	14.0 ± 1.4	N/A	0.095†
PUF-symptom	14.0 ± 4.7	16.0 ± 2.1	N/A	0.548†
PUF-bother	7.2 ± 3.4	8.8 ± 1.5	N/A	0.421†

*IC* Interstitial cystitis, *BPS* bladder pain syndrome, *VAS* visual analogue pain scale, *ICIS/ICIP* interstitial cystitis symptom index and problem index, *PUF* pelvic pain and urgency/frequency.

^†^Comparison between the IC group and BPS group.

**Figure 1:**
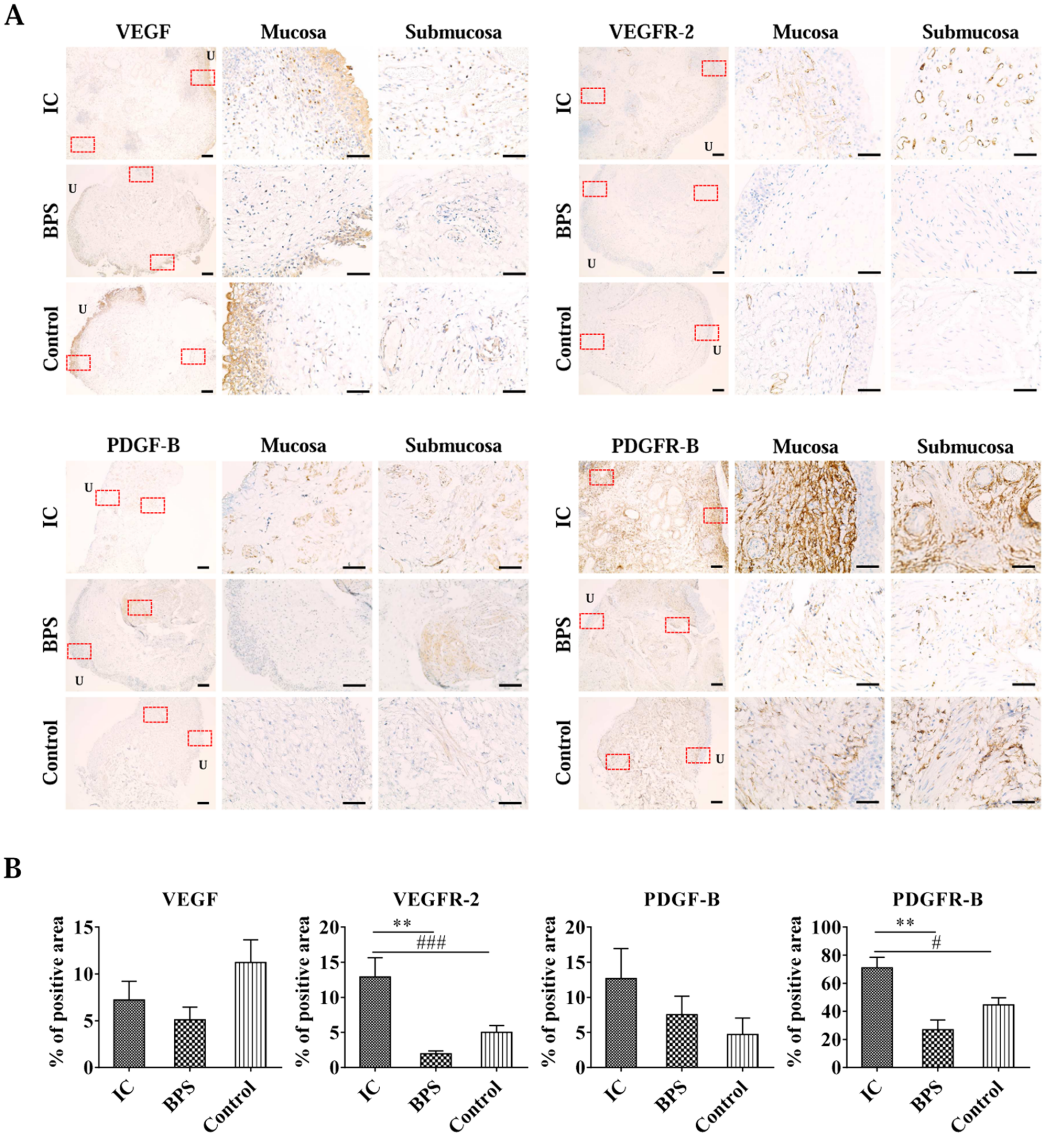
VEGFR-2 and PDGFR-B expression was increased in bladder tissues from IC patients. (**A**) Percentage of positive staining area and (**B**) representative images of each immunohistochemical staining. All data are presented as mean ± SEM, *P < 0.05, **P < 0.01, ***P < 0.001 compared with the BPS group and #*P* < 0.05, ##*P* < 0.01, ###*P* < 0.001 compared to the control with one-way ANOVA.

There was no difference in VEGF expression among groups. PDGF expression was mostly located in the lamina propria and submucosa layer; however, no statistical significance was observed between the IC group and controls. In addition, the IC group presented a more extensive VEGFR-2 and PDGFR-B positive staining area than the BPS and control group (Fig. 1a,b).

### Changes in pain threshold and voiding habitus

To investigate the therapeutic utility of axitinib, a HCl instillation-induced IC animal model in which abnormal angiogenesis was observed, similar to that in human IC/BPS bladders, was employed^11^. Pain was assessed in the animals by measuring the threshold of paw withdrawal responses to von Frey hair stimuli^12^.

**Figure 2:**
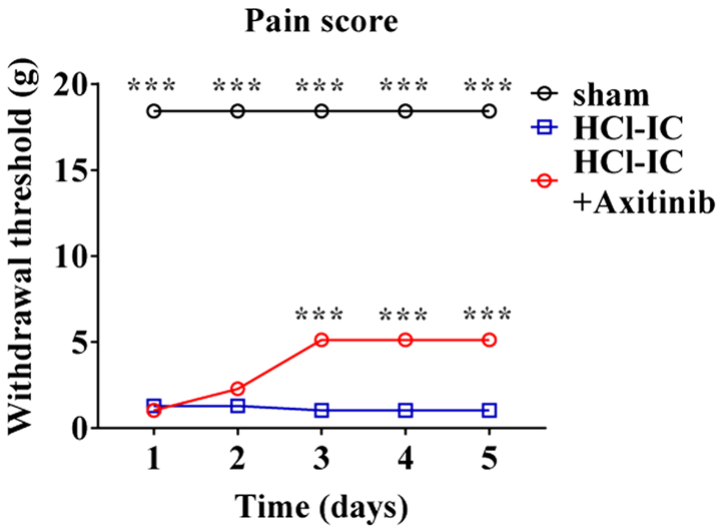
Axitinib increased the withdrawal threshold in hydrochloride (HCl)-induced interstitial cystitis (IC) rats. The withdrawal thresholds (pain score) of sham, HCl-induced IC, and axitinib-treated groups were compared using the manual von Frey test (up-down method). All data are presented as mean ± SEM, *P < 0.05, **P < 0.01, ***P < 0.001 compared with the HCl group with one-way ANOVA with Bonferroni post-tests.

The initial withdrawal threshold of the sham group was 18 g, which remained stationary from day 1 to day 5. The HCl group had a significantly lower pain threshold than the sham group. Daily administration of axitinib alleviated the susceptibility to pain; the withdrawal threshold in the axitinib group became significantly higher than the HCl group from day 3, at which point the improvement reached a plateau (Fig. 2).

**Figure 3:**
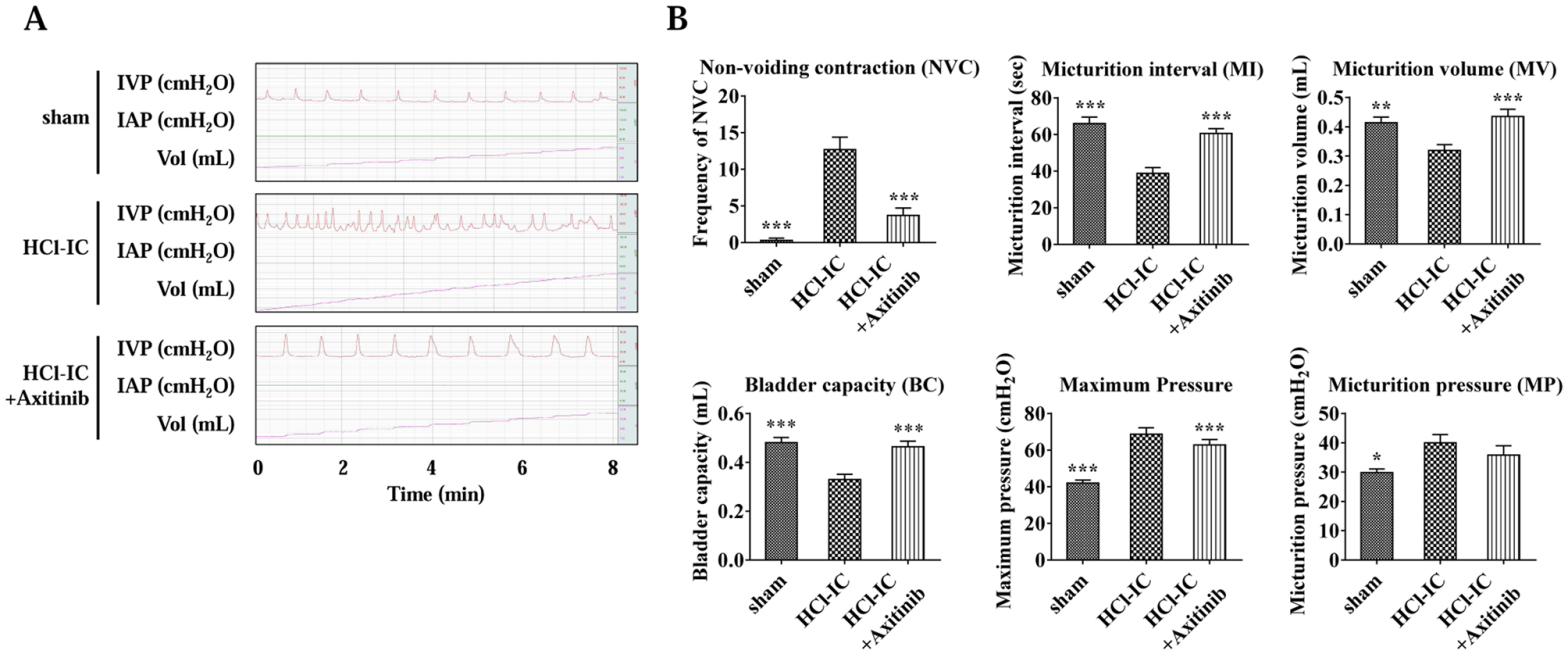
Axitinib restored bladder dysfunction in hydrochloride (HCl)-induced interstitial cystitis (IC) rats. (**A**) Representative awake cystometry results and (**B**) quantitative analysis of voiding parameters 2 weeks after HCl or saline instillation into rat bladders. All quantitative data are presented as mean ± SEM, *P < 0.05, **P < 0.01, ***P < 0.001 compared with the HCl group with one-way ANOVA with Bonferroni post-tests. (IVP; intravesical pressure, IAP; intra-abdominal pressure).

Voiding dysfunction of the HCl group was characterized by an increased non-voiding contraction, shorter micturition interval, and smaller micturition volume, resulting in a smaller bladder capacity. Oral administration of axitinib stabilized the bladder by decreasing non-voiding contraction. Furthermore, the axitinib group showed a higher micturition interval and micturition volume than the HCl group. However, there was no significant improvement in the maximum detrusor pressure or maximum intravesical pressure (Fig. 3).

### Restoration of bladder histology with and its association with angiogenesis

**Figure 4:**
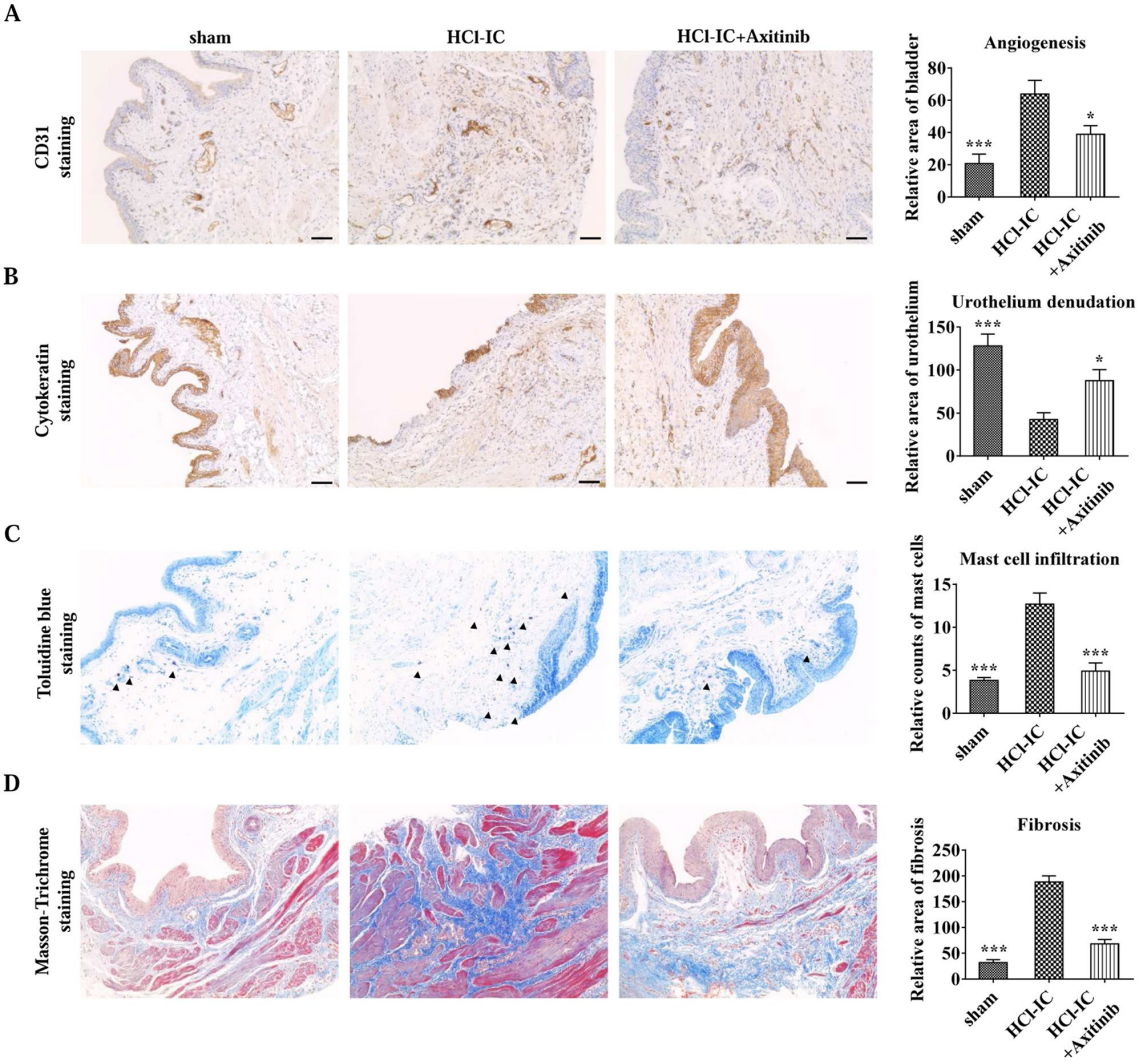
Axitinib reduced histological changes in hydrochloride (HCl)-induced interstitial cystitis (IC) rat bladders. (**A**) Representative images of cytokeratin staining and quantification of urothelial denudation (magnification × 100, scale bar = 200 μm). (**B**) Representative images of CD31 staining and quantification of angiogenesis (magnification × 200, scale bar = 200 μm). (**C**) Toluidine blue staining and quantification of mast cell infiltration (magnification × 100, scale bar = 200 μm). (**D**) Representative images for Masson’s trichrome staining (magnification × 200, scale bar = 200 μm). All quantitative data are presented as the mean ± SEM, *P < 0.05, **P < 0.01, ***P < 0.001 compared with the HCl group with one-way ANOVA with Bonferroni post-tests.

The HCl group showed more of the characteristics of IC histopathology, including included urothelial denudation, increased angiogenesis, mast cell infiltration, and fibrosis, than the sham group. Administration of axitinib alleviated HCl-induced changes in bladder histology; the urothelium was restored and angiogenesis, mast cell infiltration, and fibrosis were decreased (Fig. 4).

**Figure 5:**
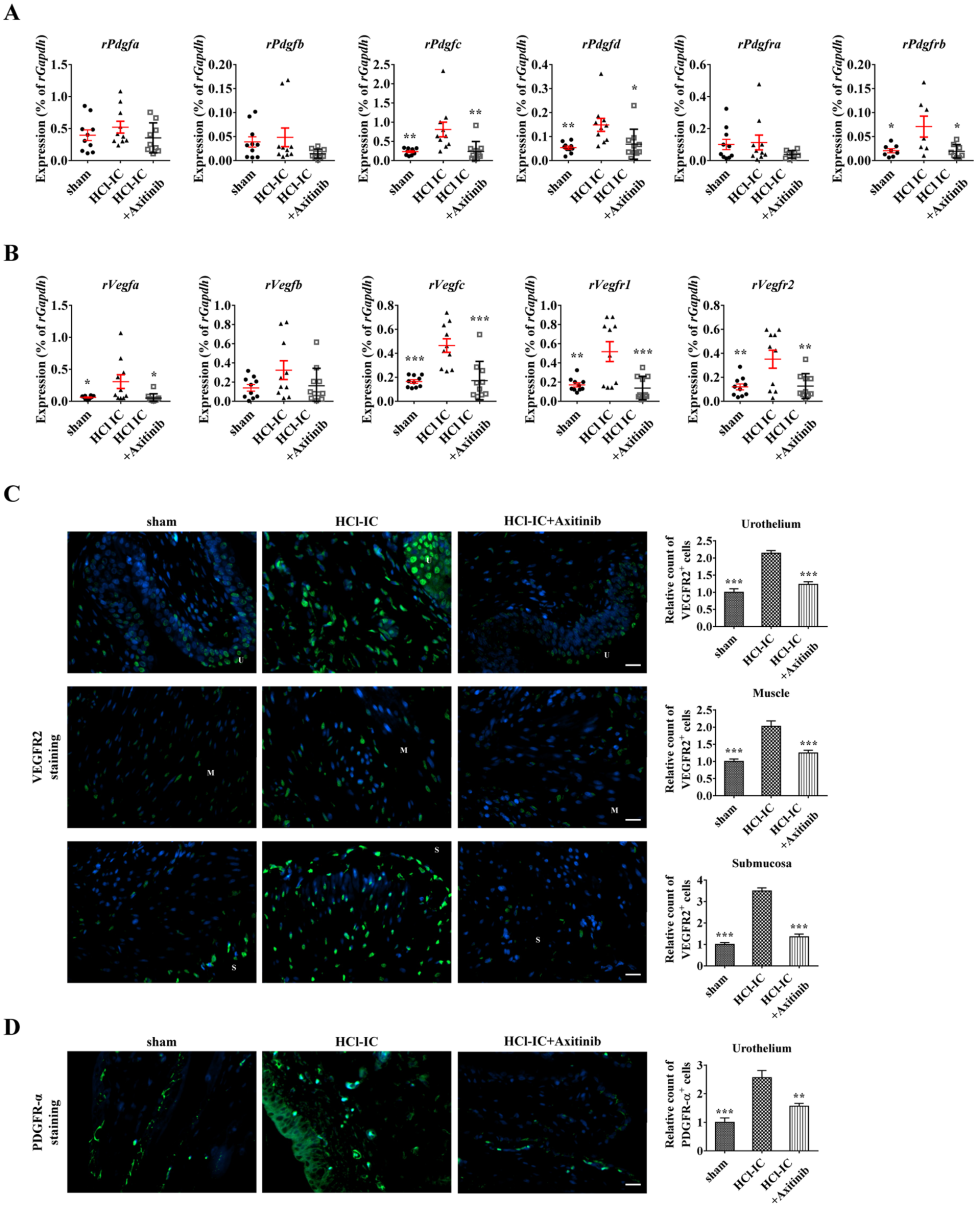
Effect of axitinib therapy on the expression of genes related to the pathogenesis of hydrochloride (HCl)-induced interstitial cystitis (IC) in rat bladders. Real-time quantitative polymerase chain reaction analysis of platelet-derived growth factor (*PDGF*) related genes (**A**), and vascular endothelial growth factor (*VEGF*)-related genes (**B**) in the indicated bladder tissues. Expression is presented as % *GAPDH* expression and shown as a dot plot of the mean and SEM (n = 10). *P < 0.05, **P < 0.01, ***P < 0.001 compared with the HCl group with one-way ANOVA with Bonferroni post-tests. (**C**,**D**) Fluorescent immunohistochemical detection of VEGFR2 and PDGFR-α protein (green) in the indicated bladder tissues (magnification × 400). Nuclei were stained with 4’,6-diamino-2-phenylindole (blue). Quantitative data of each staining are presented on the right side of the indicated representative pictures. All quantitative data were normalized to those of the sham group and are presented as the mean ± SEM (n = 5). *p < 0.05, **p < 0.01, ***p < 0.001 compared with the HCl-IC with the Bonferroni post-test.

HCl instillation increased the expression of subtypes of *PDGF* (*PDGF-C* and *PDGF-D*) and *PDGFR-B*, and peroral administration of axitinib reversed the increased expression of these genes (Fig. 5a). In addition, the upregulation of *VEGF-A*, *VEGF-C, VEGFR-1*, and *VEGFR-2* observed in the HCl model was downregulated in the axitinib group (Fig. 5b). Next, the immunofluorescence staining revealed that the level of VEGFR2 protein was increased in the urothelium, muscle, and submucosal areas of the bladders from the HCl group (Fig. 5C,D). In line with findings from IC patients, the expression of PDGFR-α protein was up-regulated in the HCl group. The treatment of axitinib ameliorated the up-regulation of these pro-angiogenic proteins.

## Discussion

In present study, we reviewed the expression of VEGF, PDGF, and their receptors in the bladder tissue of IC/BPS patients and compared them with those of healthy controls. IC patients had higher protein expression of VEGFR-2, PDGF-B, and PDGFR-B than controls. Based on these clinical findings, we investigated the therapeutic effects of axitinib, a tyrosine kinase inhibitor, in a HCl-induced cystitis rat model, which exhibits similar abnormalities in bladder function and histology to those of IC patients. In the HCl rat models, axitinib alleviated pain, abnormalities in voiding, and pathological changes through the downregulation of the mRNA expression of *VEGF, PDGF*, and their receptors, demonstrating its therapeutic potential.

Treatment strategies for IC/BPS have evolved continuously; however, pain management is the mainstay of treatment throughout therapy. In addition, it is recommended to offer a choice of therapeutic options ranging from the least to the most invasive. These stepwise approaches for IC/BPS include behavioral/non-pharmacological therapy, oral medication, instillation, and procedures, including fulguration of Hunner lesion, hydrodistension, intradetrusor botulinum toxin injection, neuromodulation, and major surgery^5^. However, the level of evidence and recommended strengths are mostly limited, except for those of behavioral/non-pharmacological therapy. Thus, there is still no definite treatment for IC/BPS.

The intractable feature of IC/BPS has stimulated robust attempts to investigate alternative therapeutic strategies including intravesical instillation of platelet-rich plasma^13,14^ and injection of stem cells into the bladder^15^. Stem cells facilitate the regeneration of damaged tissue by direct differentiation, self-renewal, and paracrine activities, which recruit various cytokines. However, there are some major issues with stem cell therapy, including determining the type, dosage, and route of administration for maximal therapeutic efficacy and safety in humans, which could require numerous trial and error studies. Testing a pre-existing medication that targets the pathogenesis of IC with a known profile might provide a more efficient and interesting option than experimental preclinical studies and clinical trials.

Angiogenesis plays an important role in the pathogenesis of various chronic inflammatory diseases. Hunner’s lesions, which distinguishes IC from BPS, are classically defined as “a circumscript, reddened mucosal area with abnormal vessels radiating towards a central scar with or without coagulum”^16^. This characteristic cystoscopic finding suggests that IC may be associated with angiogenesis. This is why we decided to study axitinib, a tyrosine kinase inhibitor with a high affinity for VEGFR 1–3, which are known to regulate angiogenesis. Previous studies investigating the angiogenic components of IC bladders reported increased expression of VEGF^6^. In addition, high expression of CD31 was reported to correlate with the severity of urinary frequency and bladder pain^7^. Another study suggested that blood perfusion is decreased in IC, especially during the filling phase, and hypoxia-inducible factor-1, a transcriptional mediator of VEGF, is also increased in IC bladders^17^.

VEGF mainly contributes to angiogenesis and lymphangiogenesis; however, it also plays a key role in bladder inflammation by promoting nerve plasticity. VEGF and its isoforms primarily act through the tyrosine kinase receptors VEGFR-1 and VEGFR-2, with VEGFR-2 being the most characterized VEGFR. The binding of VEGF to VEGFR typically results in angiogenesis (blood vessel formation) and endothelial cell proliferation until the target organ receives enough oxygen. However, in pathological conditions, VEGF secretion is not restricted resulting in the formation of immature blood vessels that are hemorrhagic and leaky^8^. A recent preclinical study reported that blockade of VEGF/VEGFR-2 signaling in rat model of acute and chronic cyclophosphamide (CYP)-induced cystitis resulted in increased bladder capacity and voided volume^18^. Similarly, imatinib, a tyrosine kinase inhibitor that inhibits PDGFR-A, -B, and C-kit, alleviated the altered expression of such receptors in rat bladders with CYP-induced cystitis^19^.

The main difference between our human IC bladder and previous studies was the expression level of VEGF. No significant difference in VEGF positive staining was observed between IC patients and controls, while the expression of VEGF was more significant in previous reports. We assume the discrepancy between references and our study results from the characteristics of the control patients and differences in disease duration. First, the control patients from the study by Kiuchi et al.^6^, were bladder cancer patients while controls in our study were healthy non-cancerous, and non-IC/BPS patients. It is impossible to perform direct one-to-one comparison between bladder tissue of non-cancerous patients and normal looking lesion of the bladder cancer patients. There is a report on VEGF expression in bladder cancer patients suggested that bladder cancer tissue has significantly higher VEGF mRNA levels than that of adjacent normal mucosa^20^. However, the VEGF expression level of tissues in bladder cancer patients are diverse; one study reported that negative VEGF expression was observed in 7.1% of bladder cancer tissue and the expression level of VEGF varied^21^. As oncological demographics of the control group is unknown, further studies on VEGF expressions in various lesions of bladder cancer patients and their direct comparison with those of non-cancerous patients are needed. Meanwhile, the control group of study by Furuta et al.^7^, consists of female patients with benign etiology such as urinary incontinence. There is a report that expression of VEGF was significantly higher among males in the age group of 50 years or older^22^. The male to female ratio of our control group is 2:3. The included male patients could have levelled-up the VEGF expression of controls.

The expression level of VEGF and its isoforms also differed between our human IC bladder and the HCl rat model. We assume this is due to the gap between the bladder injury (symptom onset) and histological evaluation. In animal models, bladder tissue was uniformly evaluated 1 week after HCl instillation, while the median gap between symptom onset and bladder biopsy was 16.3 months in the human IC and BPS groups. Acute chemical-induced bladder injury in the HCl model might have resulted in more extensive expression of VEGF, PDGF-B, and the associated receptors to restore the damaged tissue. Conversely, longer symptom duration (> 6 months) in IC/BPS patients might result in more chronic or stabilized changes in the urinary bladder. The baseline symptomatic demographics and exact diagnostic criteria of included IC patients in above references are unknown. As AUA guideline suggests the symptom duration for more than 6 months, the median gap of 16.3 months between symptom onset and bladder biopsy our study population might reflects more chronic condition.

The expression level of VEGF can be expressed either as intensity or area of positive staining. The problem with using staining intensity is inter-observer differences; thus, results might not be reproducible. In a sub-analysis of human bladder tissue based on four-scale intensities (negative: 0, mild: 1, moderate: 2, strong: 3) with manual reading of slides, there were no differences in the intensity of VEGF, VEGFR-2, PDGF-B, PDGFR-B among groups. However, three patients in the IC group had strong VEGF staining in the plasma cells adjacent to the vessels, which was not observed in the other groups. Interestingly, these patients had symptom (visual analogue scale ≥ 4) or Hunner lesion recurrence at an average of 6.8 months after transurethral resection and coagulation while another two patients without strong VEGF staining of plasma cells had no symptom recurrence at an average follow-up of 11.2 months. This finding supports the previous report that lymphoplasmacytic infiltration is more prominent in IC bladders, and that B-cell abnormalities might be involved in the pathogenesis of IC. In addition, the disease activity of IC and probability of recurrence may be associated with abnormal angiogenesis and inflammatory cell infiltration.

Whether IC and BPS are diseases from different entities remains controversial. Histological evaluation of IC/BPS has revealed that bladder tissues of Hunner-type IC patients present with severe inflammation and urothelial denudation of the entire bladder, whereas non-Hunner-type IC bladder tissues are characterized by fibrosis and increased mast cell infiltration^23^. Moreover, immunohistochemical quantification of T-lymphocytes, B-lymphocytes, and plasma cells suggested that IC and BPS are distinct pathological entities^4^. Our finding of differences in the expression of VEGFR-2 and PDGFR-B between the IC and BPS patients also supports the concept that IC and BPS are different diseases with distinct pathophysiologies.

A limitation of this study is that we analyzed bladder tissues from a small number of patients. In addition, our animal model is an acute chemical-induced cystitis rat model, which may not recapitulate the chronic nature of IC/BPS. However, we have demonstrated that axitinib has therapeutic effects in the IC rat model, which could be fundamental for the future application of axitinib in real clinical practice.

In conclusion, oral administration of axitinib improved pain, voiding profiles, and urothelial integrity through the inhibition of angiogenesis in the IC rat model. Axitinib may have potential therapeutic efficacy in IC patients based on the finding that IC bladder tissues also exhibit increased expression of VEGFR-2 and PDGFR-B.

## Supplementary Information

Supplementary Figure 1.

## Data Availability

All data generated or analysed during this study are included in this published article. Correspondence and requests for materials should be addressed to SJH or D-MS (jshinuro@ewha.ac.kr or d0shin03@amc.seoul.kr).
