# Supplementary material for: Therapeutic effects of axitinib, an anti-angiogenic tyrosine kinase inhibitor, on interstitial cystitis
Source: Sci Rep. 2023 May 23;13:8329. doi: 10.1038/s41598-023-35178-5 (PMC10205792; doi:10.1038/s41598-023-35178-5)

**Therapeutic effects of axitinib, an anti-angiogenic tyrosine kinase inhibitor, on interstitial cystitis**

Jung Hyun Shin^1†^, Chae-Min Ryu^2,3†^, Hwan Yeul Yu^2^, Yang Soon Park^4^, Dong-Myung Shin^5,*^, Myung-Soo Choo^2,*^

^1^Department of Urology, Ewha Womans University Mokdong Hospital

^2^Department of Urology, Asan Medical Center, Ulsan University College of Medicine

^3^Center for Cell Therapy, Asan Medical Center, Ulsan University College of Medicine

^4^Department of Pathology, Asan Medical Center, Ulsan University College of Medicine

^5^Department of Cell and Genetic Engineering, Asan Medical Center, Ulsan University College of Medicine

†These authors contributed equally to work.

**Supplementary Information**

**Supplementary Figure 1. Schematic description of the experiment**


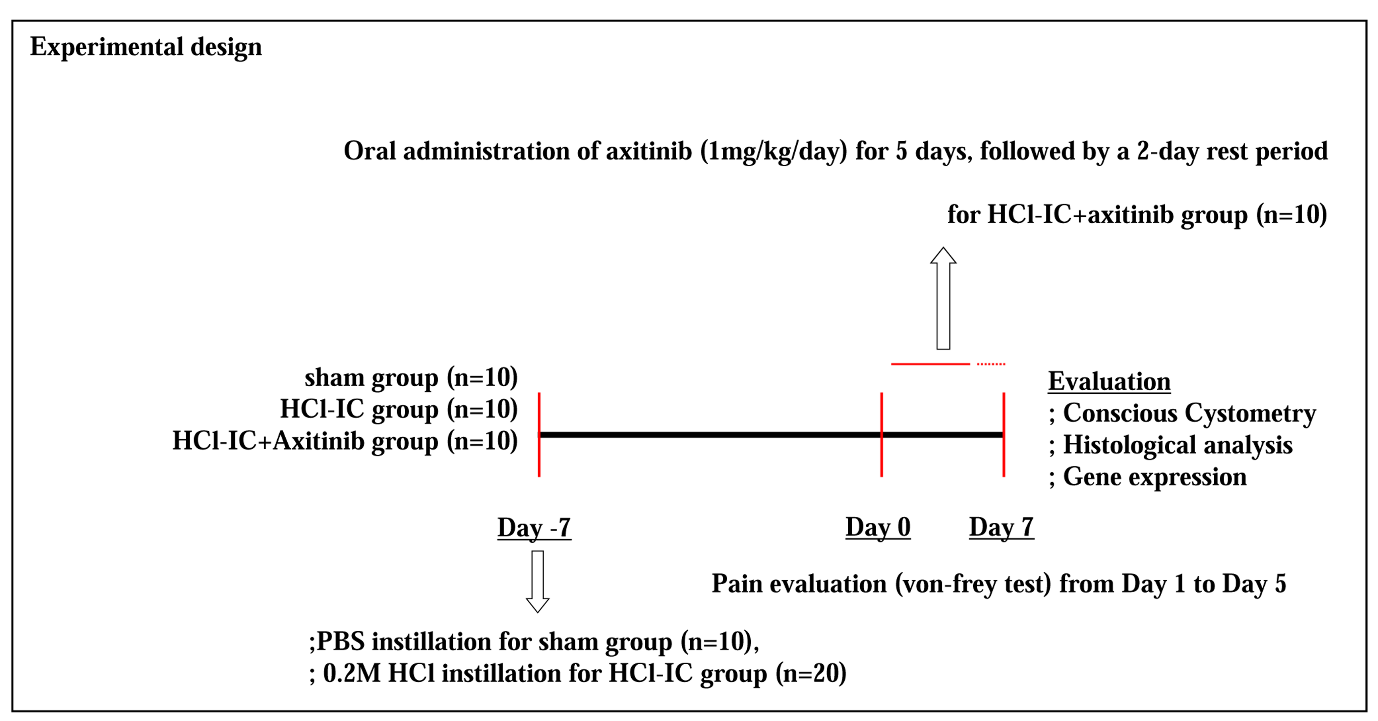

Supplement: Supplementary file 1 — Supplementary Figure 1. [file 41598_2023_35178_MOESM1_ESM.docx]
